# COVID-19: ICU delirium management during SARS-CoV-2 pandemic

**DOI:** 10.1186/s13054-020-02882-x

**Published:** 2020-04-28

**Authors:** Katarzyna Kotfis, Shawniqua Williams Roberson, Jo Ellen Wilson, Wojciech Dabrowski, Brenda T. Pun, E. Wesley Ely

**Affiliations:** 1 Department Anaesthesiology, Intensive Therapy and Acute Intoxications, Pomeranian Medical University, Al. Powstańców Wielkopolskich 72, 70-111 Szczecin, Poland; 2 Critical Illness, Brain Dysfunction, and Survivorship (CIBS) Center, Vanderbilt University Medical Center, Nashville, TN USA; 3 Department of Neurology, Vanderbilt University Medical Center, Nashville, TN USA; 4 Department of Bioengineering, Vanderbilt University, Nashville, TN USA; 5 Department of Psychiatry and Behavioral Sciences, Vanderbilt University Medical Center, Nashville, TN USA; 6 Geriatric Research, Education and Clinical Center (GRECC), Tennessee Valley Veterans Affairs Healthcare System, Nashville, TN USA; 7 Department of Anaesthesiology and Intensive Care, Medical University of Lublin, Lublin, Poland; 8 Division of Allergy, Pulmonary, and Critical Care Medicine, Vanderbilt University Medical Center, Nashville, TN USA

**Keywords:** COVID-19, Pandemic, SARS-CoV-2, Delirium, Sedation, Pain, PICS, PTSD

## Abstract

The novel coronavirus, SARS-CoV-2-causing Coronavirus Disease 19 (COVID-19), emerged as a public health threat in December 2019 and was declared a pandemic by the World Health Organization in March 2020. Delirium, a dangerous untoward prognostic development, serves as a barometer of systemic injury in critical illness. The early reports of 25% encephalopathy from China are likely a gross underestimation, which we know occurs whenever delirium is not monitored with a valid tool. Indeed, patients with COVID-19 are at accelerated risk for delirium due to at least seven factors including (1) direct central nervous system (CNS) invasion, (2) induction of CNS inflammatory mediators, (3) secondary effect of other organ system failure, (4) effect of sedative strategies, (5) prolonged mechanical ventilation time, (6) immobilization, and (7) other needed but unfortunate environmental factors including social isolation and quarantine without family. Given early insights into the pathobiology of the virus, as well as the emerging interventions utilized to treat the critically ill patients, delirium prevention and management will prove exceedingly challenging, especially in the intensive care unit (ICU). The main focus during the COVID-19 pandemic lies within organizational issues, i.e., lack of ventilators, shortage of personal protection equipment, resource allocation, prioritization of limited mechanical ventilation options, and end-of-life care. However, the standard of care for ICU patients, including delirium management, must remain the highest quality possible with an eye towards long-term survival and minimization of issues related to post-intensive care syndrome (PICS). This article discusses how ICU professionals (e.g., physicians, nurses, physiotherapists, pharmacologists) can use our knowledge and resources to limit the burden of delirium on patients by reducing modifiable risk factors despite the imposed heavy workload and difficult clinical challenges posed by the pandemic.

## Introduction

The novel coronavirus, SARS-CoV-2-causing Coronavirus Disease 19 (COVID-19), emerged as a public health threat in December 2019 and was declared a pandemic by the World Health Organization in March 2020 [1]. Many hospitalized patients with COVID-19 will develop delirium, and given early insights into the pathobiology of this virus indicating invasion into the brain stem, as well as the emerging interventions utilized to treat these critically ill patients, delirium prevention and management may prove exceedingly challenging, especially in the intensive care unit (ICU). In addition to the neurobiology of COVID-19 and typical deliriogenic factors omnipresent in the ICU, this pandemic has created circumstances of extreme isolation and distancing from human contact whenever possible, including loved ones, plus the inability to freely ambulate, which essentially create a “delirium factory” that must be explicitly addressed to maximize human dignity and respect during care.

In patients with COVID-19, delirium may be a manifestation of direct central nervous system (CNS) invasion, induction of CNS inflammatory mediators, a secondary effect of other organ system failure, an effect of sedative strategies, prolonged mechanical ventilation time, or environmental factors, including social isolation. Drawing from experience with other closely related viruses from the *coronaviridae* family, direct CNS invasion appears to occur rarely and late in the disease course but may be associated with seizures, impairments in consciousness or signs of increased intracranial pressure [2, 3]. Such symptoms may require specialized neuro-intensivist management. Immunologic responses to *coronaviridae* appear to be mediated by acute cytolytic T cell activation [4]. This response could, if dysregulated, cause an autoimmune encephalopathy [5]. Secondary effects include cerebral hypoxia or metabolic dysregulation in association with failure of pulmonary or other organ systems, such as can be seen in a variety of other types of delirium [6]. Environmental and iatrogenic factors such as prolonged mechanical ventilation, sedatives (especially benzodiazepines), and immobility also contribute heavily to the risk of ICU delirium [7] and can contribute to its development in the context of acute COVID-19 infection.

In an early retrospective report from Wuhan, Mao et al. reported that only 7.5% had any chart documentation of “impaired consciousness,” which was the only term approximating delirium [8]. Underreporting of delirium is extremely common in retrospective chart reviews, and under 1 in 10 with delirium is likely a gross underestimation. The literature is very consistent that ~ 75% of occurrences of delirium are missed in patients unless objective delirium monitoring is being employed to detect this form of acute brain dysfunction [9–15]. In addition, in COVID-19, the risk of complications such as acquired dementia and ICU-acquired weakness (ICU-AW) as well as depression and PTSD, the defining illnesses of post-intensive care syndrome (PICS), and PICS in family members (PICS-F) [16–18] will be greatly exacerbated if we allow patients to suffer unmitigated delirium.

This article will discuss how ICU professionals (e.g., physicians, nurses, physiotherapists, pharmacologists) can use our knowledge and resources to limit the burden of delirium on patients by reducing modifiable risk factors despite the imposed heavy workload and difficult clinical challenges posed by the pandemic. For example, others have already stressed reasonable analgesia and sedation use with special attention to monitoring and mitigating delirium [19].

## COVID-19: Potential factors contributing to ICU delirium

Delirium, the most frequent clinical expression of acute brain dysfunction [20], is especially important in the context of COVID-19. It may be regarded as an early symptom of infection, as previously described in septic patients [21]. Therefore, delirium should be actively screened for using dedicated psychometric tools, i.e., CAM-ICU [22] or ICDSC [23–26]. It is also plausible that delirium severity, which could be measured with CAM-ICU-7 or DRS-R-98 [27, 28], may be associated with COVID-19 severity [25, 29, 30]. The SARS-CoV-2 virus causes pneumonia, especially in elderly patients [31, 32]. Since advanced age is a well-described independent risk factor for delirium, it could be postulated that those who are at the greatest risk for severe pulmonary disease related to COVID-19 are likely at the greatest risk for delirium as well. It has been reported that nearly 90% of COVID-19 patients whose condition required admission to the intensive care unit need mechanical ventilation, either non-invasive (NIV) (42%) or invasive requiring intubation (48%) [33]. Currently, due to the reports of increased aerosolization of the viral load, NIV is not recommended yet still being used when ICU resources become limited [34].

The use of sedating medications in critically ill patients, especially sedative-hypnotics and anticholinergic agents is associated with the development of delirium [35, 36]. Despite advances in care bundles, such as the ABCDEF bundle [37–39], to reduce the incidence of delirium and improve the care of critically ill patients, recent reports from regions of the world hardest hit by COVID-19 suggest that a flexible approach to management algorithms may be required, due to either a strained workforce or scarcity of resources [40]. Highlighting the importance of COVID-19-related morbidities it must be underlined that agitation associated with hyperactive delirium could theoretically be a source of intra-hospital disease spread in uncooperative patients in over-crowded settings with respiratory distress prior to intubation or awaiting admission to the ICU.

**Fig. 1:**
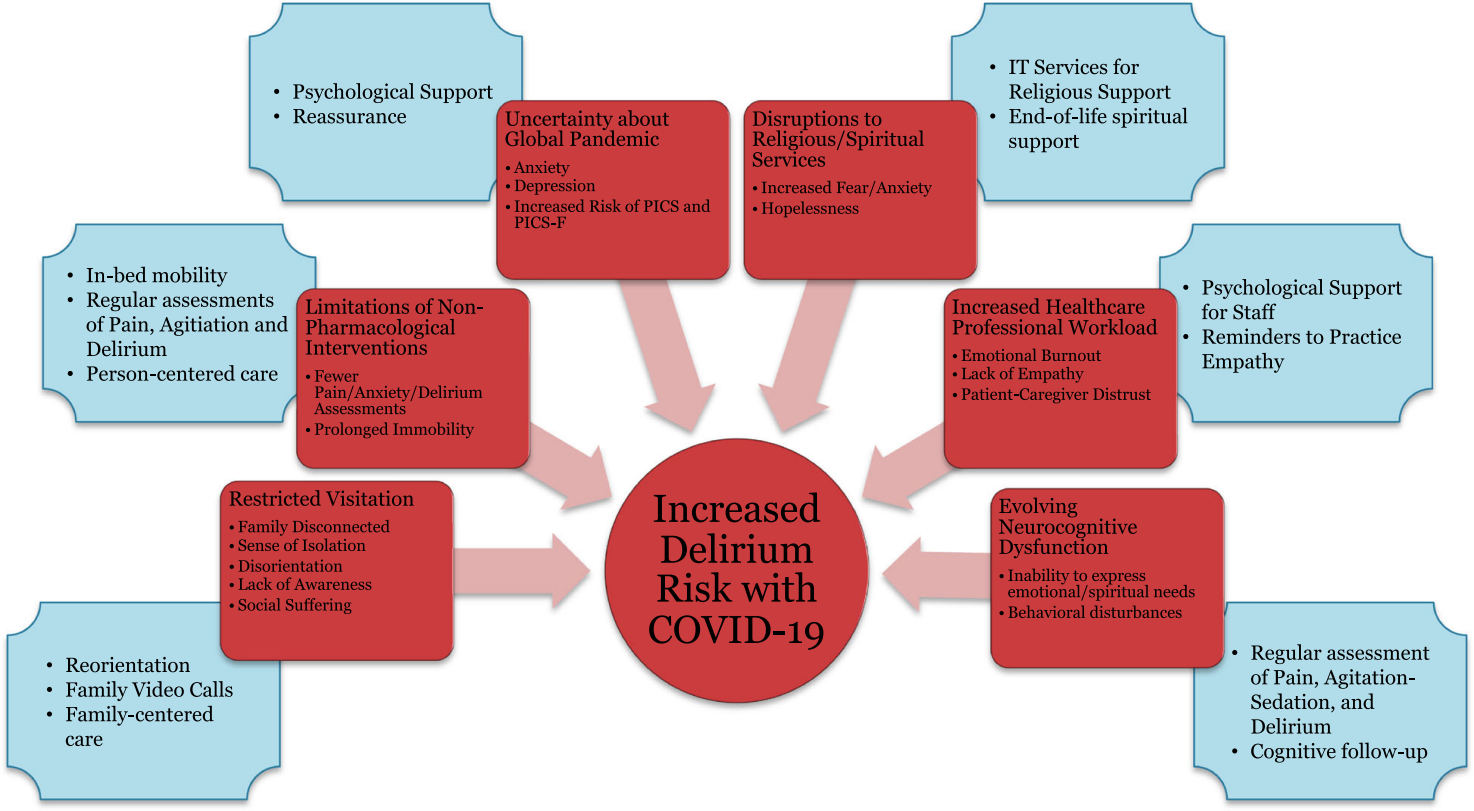
Potential factors contributing to ICU delirium during the SARS-CoV-2 pandemic

Another potential factor contributing to the occurrence of ICU delirium during the SARS-CoV-2 outbreak is social isolation created by “social distancing” strategies and quarantines, which may prove especially difficult in older adults, who have no or limited support from caregivers. In the age of COVID-19, in an attempt to “flatten the curve” and slow the spread of the virus, many hospitals have instituted no-visitation or very limited visitation policy, which may propagate a sense of isolation, ultimately contributing to disorientation and lack of awareness in the patient. What is needed now, is not only high-quality ICU care, concentrated on providing adequate respiratory support to critically ill patients, but an identification of the source and degree of mental and spiritual suffering of patients as well as their families to provide the most ethical and person-centered care during this humanitarian crisis. Many patients, coming from different religious backgrounds, will need the support of religious services that are likely to be unavailable for an extended period of time. Implementation of policies that prevent visitors from coming into the hospital should be followed by additional efforts to support patient-family interaction. This must include dedicated time and effort for phone and video conversations during busy ICU time. Moreover, hospital management should provide all possible novel technological options for communication, including teleconferences or portable speakerphones. All of these concepts are summarized in Fig. 1.

This patient-centered approach is especially important for delirious patients, the majority of whom are elderly, may suffer from an evolving neurocognitive disorder, be hypoactive or aphasic and cannot express their emotional or spiritual needs, and would typically receive comfort from relatives, friends, and caregivers, during a medical crisis. During these strenuous and difficult times, an even deeper sense of humanity is required from healthcare professionals and hospital management to provide quality care to critically ill patients. The workload is already increased with the volume of new and deteriorating patients, but in order to provide maximum humanitarian care and preserve the sense of dignity, we must view the fulfillment of mental and spiritual needs as a medical intervention. Yet it is obvious that during the COVID-19 pandemic, the potential for non-pharmacological interventions encapsulated in the ABCDEF bundle (e.g., mobility outside the ICU room, family engagement) may be extremely limited [26]. All of these issues factor into the type of survivorship that our COVID-19 patients and their families will experience the months and years ahead as they face the burdens of PICS and PICS-F [16–18].

## COVID-19: Neuro-invasive potential of SARS-nCoV-2 as cause of delirium

Acute brain dysfunction, symptomatically presenting as delirium (also called encephalopathy), may be a feature of the neuro-invasive potential of SARS-CoV-2. Neurotropism of *coronaviridae* has been demonstrated during SARS and MERS epidemics [41–43]. During the 2002–2003 SARS epidemic older subjects presented not only with respiratory symptoms and typical febrile response, but also with decreased general well-being, poor feeding, and delirium [44]. Given the fact that SARS-CoV and SARS-CoV-2 are similar in terms of pathogenicity, it is quite likely that SARS-CoV-2 has a similar ability to cause delirium [45].

Most CoVs share a common viral structure, infection potential, and neurotropism [46, 47], CoVs are large, enveloped viruses with a large positive-sense, single-stranded RNA genome [48]. Human pathogenic CoVs include those causing recent epidemics, severe acute respiratory syndrome CoV (SARS-CoV and SARS-CoV-2), Middle East respiratory syndrome CoV (MERS-CoV) HCoV-229E, and other identified coronaviruses, i.e. HCoV-OC43, HCoV-NL63, and HCoV-HKU1 [49–51]. CoVs have been associated with CNS diseases such as acute viral encephalopathy, acute disseminated encephalomyelitis, and multiple sclerosis and are increasingly recognized as presenting a neurologic crisis [3, 52, 53]. In one series of 183 children hospitalized with acute encephalitis, 12% were associated with coronavirus infection [54]. Such propensity of CoVs has been documented for several of the beta-CoVs, including SARS-CoV and MERS-CoV [46, 48]. Acute necrotizing encephalitis has also been described in one case of SARS-CoV-2. The patient presented with fever, cough, and altered mental status and was found to have hemorrhagic rim enhancing lesions in the deep gray matter of the cerebral cortex bilaterally [55]. Animal studies suggest coronaviruses are delivered through the peripheral nerves and may access the central nervous system through retrograde synaptic transmission [43, 56, 57]. SARS-CoV spreads in the brains of intranasally inoculated mice primarily via the olfactory bulb with subsequent infection of the hypothalamus and brainstem [57]. Such neuro-invasive potential of SARS-CoV-2 has been postulated to contribute to respiratory failure observed in infected patients [45].

The exact mechanism for neurotoxicity may depend on the brain entry route, which has not been fully elucidated [48]. The SARS-CoVs enter human host cells mainly via a cellular receptor angiotensin-converting enzyme (ACE2), expressed not only in the entire respiratory tract (which it destroys resulting in the leading cause of death), but also in the upper esophagus or enterocytes and showing very low expression level in the brain under normal conditions [45]. The virus entry route may be respiratory, via oro-fecal route, but also directly intranasal [42, 48, 58]. The possible brain entry routes for CoVs, including SARS-CoV-2, include either direct intranasal access to the brain via olfactory nerves (with anosmia as an early symptom) or indirect access to the brain by crossing the blood-brain barrier (BBB) via hematogenous or lymphatic spread [2, 41, 59].

There are several mechanisms of coronavirus-related brain damage. One of them is connected with the dysfunction of renin-angiotensin system in the brain. ACE is the major component of the cerebral renin-angiotensin system and is localized in the endothelia of cerebral vasculature [60]. The use of ACE inhibitors for treatment of blood hypertension reduces cognitive dysfunction through their anti-inflammatory actions [61]. Circulating renin-angiotensin components do not affect the brain with airtight BBB. However, general inflammatory response to virus infection impairs BBB integrity leading to massive infiltration of renin-angiotensin components to the brain [62]. Uncontrolled infiltration of the brain with renin-angiotensin components induces neuroinflammatory cascades resulting in extensive neurodegradation followed by cognitive dysfunction [63]. The SARS-CoVs can enter the brain via the BBB angiotensin-converting enzyme receptors and induce neurodegeneration, astrogliosis, and neuroinflammation. It is noteworthy that SARS-CoV particles have been found in the brain [41, 42].

Inflammatory response of the CNS to viral infection seems to be another important reason for poor neurological outcome and occurrence of delirium. A few hours after infection, neutrophils and monocytes infiltrate CNS, and neutrophils seem to be crucial in disruption of BBB permeability [64–66] The postmortem study documented a massive infiltration of the brain by immune cells, which was associated with neuronal edema and the scattered red degeneration [42] Noteworthy, activated macrophages and microglia have been present in areas of demyelination and play a critical role in myelin destruction [66]. The hypomyelinated axons were found in experimental animals with short- and long-term memory deficit, and the degree of myelin disorders was associated with memory dysfunction and short- and long-term cognitive dysfunction [67, 68]. A large amount of damaged myelin following neuroinflammation is potentially immunogenic and activates macrophages again, which initiate a vicious cycle sustaining further inflammation. This prolonged inflammation may be responsible for the higher incidence of neuropsychological abnormalities in patients with severe infection and sepsis; however, this hypothesis should be confirmed in further studies.

The median time from the onset of first symptoms to the diagnosis of respiratory compromise (dyspnea) is usually 5 days and 8 days from admission to the intensive care unit with severe respiratory failure requiring intubation and mechanical ventilation [45]. The latency period indicates that there might be sufficient time for the coronavirus to enter and destroy CNS neurons.

Previous studies have shown that some patients infected with SARS-CoV-2 present with neurological symptoms such as headache (about 8% of cases) or centrally mediated nausea and vomiting (about 1% of cases) [50, 69]. A retrospective study performed by Mao et al., reporting data from 214 COVID-19 patients, showed that neurological symptoms were present in 45% of severely ill patients, with symptoms including both acute cerebrovascular disease and impaired consciousness [8]. Possibly the neuro-invasive potential of SARS-CoV-2 may be associated with centrally mediated respiratory failure. As a hypothesis, early identification of patients with delirium, being an early symptom of CNS involvement is critical in COVID-19 patients, as it may indicate impending respiratory failure due to the neuro-invasive potential of SARS-CoV-2.

## COVID-19: ICU delirium management—potential problems and solutions

Historically, delirium rates among mechanically ventilated ICU populations were consistently 70–75%, and the duration of delirium has consistently proven an independent predictor of longer lengths of stay, higher mortality, greater cost of care, and alarming rates of acquired dementia that lasts years following illness [70–73]. Given these facts, it is important to carry into the pandemic the knowledge that delirium in mechanically ventilated patients can be reduced dramatically to 50% using a culture of lighter sedation and mobilization via the implementation of the safety bundle called the ABCDEFs promoted by the Society of Critical Care Medicine (SCCM) in their ICU Liberation Collaborative [37, 38]. Limitations in the ability to conform to this approach are a major component of the burden of the isolation required to limit the spread of COVID-19, prompting us to discuss specifics related to bedside care that one might keep in mind in organizing busy triage units and routine ICU care during the pandemic.

Delirium screening only takes 30 s. As such, delirium screening and treatment should follow well-established international guidelines, such as the eCASH concept [74] and the SCCM clinical practice guidelines [26]. Although routinely used in clinical practice, some sedation- and delirium-associated issues may be especially important when using limited resources. Standard non-pharmacological measures to treat or prevent delirium may not be possible in isolation environments, and these environments may themselves worsen delirium. Pain management remains a priority for all patients and requires the widespread implementation of behavioral pain scales (CPOT or BPS) for sedated and mechanically ventilated patients. After pain control is adequately assured, we must focus on the intersecting issues that lead a person’s brain to fail in critical illness, chief among them including overuse of powerful sedatives and undue immobilization. These and other potential problems regarding ICU delirium management during the SARS-CoV-2 pandemic are identified in Table 1.

**Table 1:** COVID-19 delirium management considerations via SCCM’s ABCDEF safety bundle framework

	Feature	Potential problem during COVID-19 Pandemic	Potential solutions
**A**	**A**ssessment/treatment of pain	Although regarded as a priority, in intubated, deeply sedated patients, assessment and management require the use of behavioral pain scales that may at first glance seem burdensome for strained healthcare workers but which will ultimately provide the most humane care and help reduce PTSD.	Regular pain assessment (NRS, CPOT/BPS)—especially in prone position. Provide adequate pain management, identify uncommon sources of pain. Consider development of peripheral neuropathies from viral invasion of peripheral nerves and PICS-related complications.
**B**	**B**oth SAT and SBT	Stopping both sedation and the ventilator to conduct daily spontaneous awakening trials and spontaneous breathing trials is essential. These will not be possible during paralysis in proned patients, which creates a serious risk-benefit choice of this modality of patient positioning that argues for the shortest duration possible. Precautions for early extubation must be used to lower the spread of aerosol	For patients who need NMBD infusion (paralyzed patients)—monitor NMB depth and shorten duration whenever possible. Regularly assess patients with both SBT and SAT daily.
**C**	**C**hoice of sedation	Sometimes, deep sedation may be necessary, especially when using NMBD, when providing high PEEP, and when prone positioning is implemented. GABA-agonist propofol is likely the best choice during proning, but this can be shortened via daily questioning of the necessity of this management approach	Assess with RASS/SAS regularly. Adjust sedation to ventilation needs—priority lies in effective ventilation (RASS-4 for prone position). As soon as possible, discontinue potent sedatives or use those agents that do not suppress the respiratory drive such as intermittent use of antipsychotics or alpha-2 agonists. Remember prolonged ventilation is associated with poor outcomes.
**D**	**D**elirium	Hyperactive delirium and agitation can be a source of intra-hospital cross-infection, especially in agitated patients or during non-invasive ventilation (if used, not recommended). Hypoactive delirium is likely to be missed if not monitored for using a validated instrument routinely. Thus, patients may not receive appropriate attention to delirium prevention mechanisms.	Provide regular delirium screening (CAM-ICU, ICDSC). Provide usual non-pharmacological interventions: (1) orientation is a priority, because patients see healthcare wearing personal protective equipment; (2) support for senses (hearing aids/glasses); (3) monitor taste/smell failure due to CoV predilection to olfactory nerves (anosmia may be an early sign). Limit the use of CNS-active medications to agitated patients. When CAM-ICU or ICDSC positive, use the **Dr. DRE** mnemonic to consider chief delirium risks: **D**iseases (new nosocomial infections, acquired heart failure); **D**rug **R**emoval, stop all unnecessary psychoactive medications, be on the lookout for withdrawal if the patient was on a prolonged course of sedatives; **E**nvironment, maximize sleep, orientation to other humans, minimize sensory deprivation.
**E**	**E**arly mobility	Physiotherapy may be very limited due to heavy workload and epidemiologic precautions; infusion of NMBD may be necessary.	Physiotherapy must be adjusted to heavy workload and epidemiologic precautions. Use passive physiotherapy interventions during the infusion of NMBD.
**F**	**F**amily presence	Limited or no family presence during the pandemic due to quarantine and social distancing. A major issue for elderly and as end-of-life problem.	Orientate both patients and family regularly, provide phone conversations and video conferences, use technology devices, headphones, and tele-medicine tools. Provide visual and vocal contact with the family/caregivers/friends, especially for all dying patients despite isolation, lack of time, and heavy workload.

*BPS* behavioral pain scale, *CAM-ICU* cognitive assessment method for intensive care unit, *CNS* central nervous system, *CoV* coronavirus, *CPOT* critical pain observation tool, *ICDSC* intensive care delirium screening checklist, *NMBD* neuromuscular blocking drugs, *NRS* numeric rating scale, *PEEP* positive end-expiratory pressure, *PICS* post-intensive care syndrome, *SAT* spontaneous awakening trial, *SBT* spontaneous breathing trial

During such harrowing times at the respiratory failure that is occurring with COVID-19, it would be easy to disregard patients’ brains as not being an essential concern. If we follow the critical care literature, this would be a grave error. Evidence indicates that delirium is not only a robust prognostic indicator of worse survival immediately, but also of the cost of care and quality of survival [71–73, 75, 76]. Thus, healthcare professionals should follow local guidelines and policies regarding the monitoring and management of delirium. Implementation of easy screening methods for delirium is necessary especially in light of heavy workload because without validated assessment tools 75% of delirium will be missed [9–15, 24] during the COVID-19 crisis. It is necessary to reduce the ICU delirium risks using standard management approaches towards adequate pain management, avoiding urinary retention and gastro-intestinal problems (constipation), identification and treatment of nosocomial sepsis, and maintaining adequate oxygenation. Non-pharmacological interventions such as regular orientation despite social separation and lack of contact with family and caregivers are going to prove vitally important.

Regarding pharmacological interventions, no drugs can be recommended for the prevention or treatment of ICU delirium other than avoidance of overuse of potent psychoactive agents like sedatives and neuromuscular blockers (NMB) unless patients absolutely require such management [77–80]. This component of the conversation is especially important given the early anecdotal recommendations to treat patients with COVID-19 in the prone position [81], which will be uncomfortable and thus likely be met with even higher than usual amounts of sedation, which could beget very high rates of delirium down the line in the management of these already high-risk patients. Additionally, it is important to review previous medications to avoid withdrawal symptoms. The ease of COVID-19 transmission and the risk of harm to others (healthcare workers, family, caregivers) may exceed risk of harm to the individual. This is an isolated example warranting earlier use of sedatives for hyperactively delirious patients who are proving harmful to self and others. ICU beds and ventilators are valuable and needed resources so it will be important to consider ways to avoid unnecessary prolongation of ventilation time and ICU length of stay that is associated with deeper sedation. Table 1 provides an overview of ABCDEF bundle adaptations to meet the needs of COVID-19.

## Conclusions

Data regarding delirium in the SARS-CoV-2 pandemic era are thus far too limited. This virus destroys the respiratory tract and invades the CNS, both of which will produce an extremely high-risk circumstance for both acute and long-term brain dysfunction in patients infected with the COVID-19 virus. The further elements of human isolation, extended time away from family and other loved ones, and other elements of care all form what could be construed as a delirium factory that medical teams must address. In the patients with COVID-19, delirium can be a manifestation of direct CNS invasion, induction of CNS inflammatory mediators, secondary effects of other organ system failure, and untoward medical and environmental factors including heavy use of sedatives for prone positioning of the patient and quarantining and social isolation during care. The focus during the COVID-19 pandemic obviously lies within the dire necessity of organizational issues, i.e., lack of ventilators, shortage of personal protection equipment, resource allocation, prioritization of limited mechanical ventilation options, end-of-life care. It is precisely during such times that standardization of safety concerns encapsulated in the ABCDEF bundle can provide a framework to help us accomplish “whole person” care that will help with acute management success as well as improvement of long-term survivorship and reductions of PICS and PICS-F burden on individuals and society as a whole. At the heart of this safety bundle lies the brain, the most vital organ of the human body, and it has been shown now in over 25,000 patients [37, 39, 82, 83] that higher compliance yields better survival, less delirium and coma, shorter lengths of stay, less ICU bounce-back, and lower cost of care. Implementation at the bedside of excellent delirium prevention and management should be a priority during the COVID-19 pandemic [84, 85].

## Data Availability

Not applicable.
